# Anti-mutagenic agent targeting LexA to combat antimicrobial resistance in mycobacteria

**DOI:** 10.1016/j.jbc.2024.107650

**Published:** 2024-08-08

**Authors:** Chitral Chatterjee, Gokul Raj Mohan, Hariharan V. Chinnasamy, Bhumika Biswas, Vidya Sundaram, Ashutosh Srivastava, Saravanan Matheshwaran

**Affiliations:** 1 Department of Biological Sciences and Bioengineering, Indian Institute of Technology Kanpur, Kanpur, Uttar Pradesh, India; 2 Department of Biological Sciences and Engineering, Indian Institute of Technology, Gandhinagar, Gujarat, India; 3 Centre for Environmental Sciences and Engineering, Indian Institute of Technology Kanpur, Kanpur, Uttar Pradesh, India; 4 Mehta Family Centre for Engineering in Medicine, Indian Institute of Technology, Kanpur, Uttar Pradesh, India; 5 Kotak School of Sustainability, Indian Institute of Technology, Kanpur, Uttar Pradesh, India

**Keywords:** *Mycobacterium tuberculosis*, SOS response, antimicrobial resistance (AMR), LexA, small molecule inhibitor, transcription

## Abstract

Antimicrobial resistance (AMR) is a serious global threat demanding innovations for effective control of pathogens. The bacterial SOS response, regulated by the master regulators, LexA and RecA, contributes to AMR through advantageous mutations. Targeting the LexA/RecA system with a novel inhibitor could suppress the SOS response and potentially reduce the occurrence of AMR. RecA presents a challenge as a therapeutic target due to its conserved structure and function across species, including humans. Conversely, LexA which is absent in eukaryotes, can be potentially targeted, due to its involvement in SOS response which is majorly responsible for adaptive mutagenesis and AMR. Our studies combining bioinformatic, biochemical, biophysical, molecular, and cell-based assays present a unique inhibitor of mycobacterial LexA, wherein we show that the inhibitor interacts directly with the catalytic site residues of LexA of *Mycobacterium tuberculosis* (Mtb), consequently hindering its cleavage, suppressing SOS response thereby reducing mutation frequency and AMR.

The silent pandemic of antibiotic resistance is on the rise. Nearly 1.27 million people succumb to AMR-related deaths every year (1). Out of the 7.7 million deaths from bacterial infections, 1.6 million result from tuberculosis (TB) alone, caused by *Mycobacterium tuberculosis* (Mtb). This is a major cause of concern as drug-resistant TB cases increased by 3% within a span of 1 year (2020*–*2021) (2). There is a 13% increase in total TB cases and a 32% increase in multidrug-resistant/rifampicin-resistant TB cases in India, annually (3). Without better treatment and control strategies, insurmountable losses will be incurred in the future. The emergence of extensive multidrug-resistant strains has complicated the prospects of controlling and eliminating TB (4). Therefore, an effective strategy could be to target the proteins involved in regulating the pathways that mediate mutagenesis by developing anti-mutagenic molecules (5).

The concerted action of DNA damage repair pathways and the SOS response accounts for the increased mutability, adaptability, and emergence of drug-resistant pathogens (6, 7, 8, 9). Over the last decade, efforts aimed at targeting the SOS response have been gathering momentum to strengthen therapeutic efficacy (10, 11, 12). Blocking the SOS response impairs the proficiency of the bacteria to repair its damaged DNA in response to stress. Moreover, error-prone mutagenesis in response to SOS activation, which further potentiates the development of drug-resistant mutations, can be avoided by directly inhibiting the activation of this pathway (13, 14). Screening and characterization of SOS inhibitors can help us in targeting the stress adaptation DNA repair-mutagenesis axes of Mtb. This can help in augmenting the already existing drug regimen and act as an adjuvant therapy to counter multidrug resistance.

SOS response in bacteria is regulated by two master regulator proteins- LexA (transcriptional repressor, which upon DNA damage undergoes autoproteolysis to activate the SOS pathway) and RecA (activator, which catalyzes the autoproteolysis of LexA) (15, 16, 17, 18, 19, 20, 21). Inactivating the master-regulator proteins controlling this pathway has been shown to cause decreased mutagenesis post-antibiotic treatment, decreased minimum inhibitory concentration (MIC) of DNA-damaging antibiotics, and re-sensitization of drug-resistant strains (8, 22, 23). Inhibitors of RecA from both *Escherichia coli* and Mtb have been identified (7, 10, 24, 25, 26). For example, suramin has been demonstrated to be a potent inhibitor of bacterial RecA proteins, augmenting the antimicrobial properties of ciprofloxacin (7). Phthalocyanine tetrasulfonic acid compounds active against bacterial RecA, have been shown to, inhibit the SOS response and reduce mutation frequency in both Gram-positive and Gram-negative bacteria (25). Another promising strategy involved the usage of synthesized short peptides based on RecX (inhibitor of RecA) structure, which displayed SOS response inhibition *in vivo* and hindered the functions of RecA *in vitro* (10). High-throughput screening with ∼34,000 compounds resulted in the identification of four chemotypes that displayed potential RecA inhibitory activity (26). However, further characterization is required to assess the therapeutic potential of the drugs belonging to these chemotypes. RecA inhibition in bacteria depleted of DNA gyrase results in a reversion of persistence and enhanced efficacy of antibiotics (27, 28). Homologs of RecA are known to exist in most prokaryotic and eukaryotic organisms which makes it challenging to target RecA. Recently, efforts have been undertaken to target the other master regulator, LexA, which is absent in eukaryotes, making it a potential target (8, 29, 30, 31, 32, 33). An extensive collaborative study resulted in the identification of novel inhibitors targeting *E. coli* LexA autoproteolysis (8). Since LexA plays a crucial role in the SOS response, inhibitors of Mtb LexA would directly target the mutagenesis and drug resistance axes. Recently, 3-aminophenyl boronic acid (3-aPBA) has been reported to inhibit the autoproteolysis of *E. coli* LexA (29). The kinetics of interaction between *E. coli* LexA and 3-aPBA and the anti-mutagenic potential of the molecule had not been studied. Further insights into the mechanistic aspect needed probing to understand the molecular mechanism. Presently, no known inhibitors of Mtb LexA have been identified and Mtb LexA differs from its *E. coli* counterpart in several ways (34). Mtb LexA harbors additional stretches of amino acids at its N-terminal and linker region (35). Unlike its *E. coli* counterpart, Mtb LexA interacts with different SOS box sequences with comparable nanomolar affinity *in vitro* (34). Since the boronic acid class of inhibitors has been reported to block the catalytic activity of the serine protease family of proteins, we hypothesized that this class of inhibitors could exert a similar effect on Mtb LexA as it belongs to the same family of proteins (15, 36). Through computational analysis, biochemical, biophysical, molecular, and cell-based assays, we identified a potential inhibitor of Mtb LexA which was found to be effective in preventing its autoproteolytic cleavage, resulting in stalling of the SOS response. Further, we also observed a stark decrease in the mutation frequency of mycobacterial cells and the down-regulation of important genes under the SOS regulon. This study reveals the inhibition of the mycobacterial SOS pathway by a newly identified inhibitor. Essentially, it holds promise as an anti-mutagenic agent that may strengthen the current arsenal to boost anti-TB therapeutic strategies.

## Results

### Screening of compounds to identify a potential inhibitor of mycobacterial SOS response

**Figure 1:**
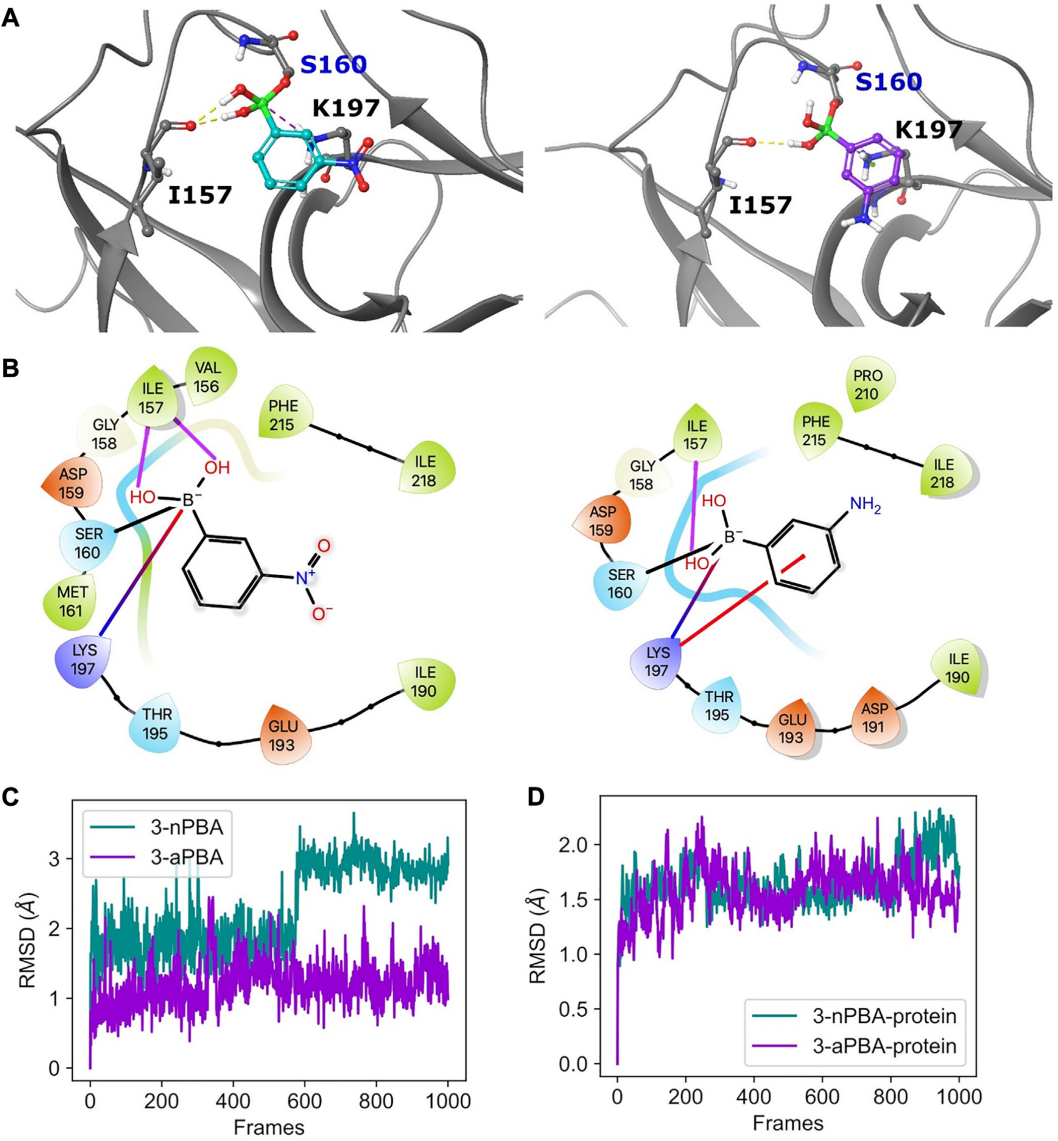
**Interactions and stability of covalently docked 3-nPBA and 3-aPBA.***A*, docked pose of covalently bonded 3-nPBA (*left*) and 3-aPBA (*right*) in the binding pocket of Mtb LexA. Protein is shown in the *gray* cartoon representation, while the ligands are shown in the ball and stick representation. The interacting residues are represented in *gray lines*, and reactive residue S160 is in *gray* ball and stick representation. Electrostatic interactions and Hydrogen bonds are shown as *yellow* and *red* spotted lines, respectively. *B*, 2D ligand interaction figure of 3-nPBA (*left*) and 3-aPBA (*right*). Hydrogen bonds are shown in *purple arrows*, electrostatic interaction is shown in a *blue-red* shaded line, and cation-pi interaction is shown in as *red line*. *C*, root Mean Square Deviation (RMSD) of 3-nPBA and 3-aPBA during 10 ns (1000 frames) run. *D,* RMSD of protein Cα atoms of 3-nPBA and 3-aPBA docked complexes during 10 ns (1000 frames) run.

To identify a potential Mtb LexA inhibitor, we first did molecular docking and then molecular dynamics (MD) simulations. Considering that boronic acid-based compounds have previously demonstrated inhibition of *E. coli* LexA (29), we evaluated several compounds for their binding to Mtb LexA, including three FDA-approved drugs (Table S1). The rationale for selecting these compounds was that the boron moiety displayed a covalent interaction with the hydroxyl group of a serine residue in the active site of serine hydrolases. We predicted this mechanism might apply to Mtb LexA, a known serine protease (36, 37). First, we performed non-covalent docking of these compounds at the active site of Mtb LexA (see Supporting information). All compounds exhibited low docking scores, indicating weak binding (Table S1). To further test the stability of these compounds in the active site, we performed molecular dynamics simulations, which revealed the instability of these compounds leading to their exit from the binding pocket (Fig. S1*A*). To circumvent this issue, we employed the covalent docking approach to assess the compounds’ ability to remain inside the protein’s binding pocket, having formed the covalent interaction. The compounds displayed varying covalent binding affinities and Molecular Mechanics Generalized Born and Surface area solvation (MMGBSA) scores (Table S1). We focused our analysis on 3-nitrophenyl boronic acid (3-nPBA), which displayed the most negative MMGBSA score and a high covalent docking affinity, as well as 3-aPBA, a compound previously shown to inhibit *E. coli* LexA (29). The catalytic site residues (S160, K197) involved in the autoproteolytic activity of Mtb LexA served as the predicted binding sites for 3-nPBA, indicating that it may act by preventing the autoproteolytic activity of Mtb LexA, thereby disallowing the repressor from dissociating from the cognate SOS boxes of LexA-regulated genes. As shown in Figure 1, the boronate oxygen atoms of 3-nPBA and 3-aPBA could be involved in forming hydrogen bonds with the carbonyl oxygen of I157. Moreover, the phenyl ring π ring electrons of 3-aPBA could possibly interact through cation-π interactions with K197 and boron could make ionic interactions with K197 in 3-nPBA (Fig. 1, *A* and *B*). The above-mentioned interactions with boronate oxygen atoms and phenyl rings of boronic acid derivatives have been observed previously (38).

To understand the stability of these covalently bound compounds, we performed MD simulations of these complexes. Both the compounds showed stable binding in the pocket throughout simulations (Fig. 1, *C* and *D*). The interactions of 3-nPBA and 3-aPBA during the simulations showed very similar interactions. Most importantly, the compounds show ionic interactions with the catalytic K197 residue (Fig. S1). We then examined the binding energies of the two compounds during the simulations. 3-nPBA showed more negative binding energy (−29.9 ± 4.1 kcal/mol) as compared to 3-aPBA (−23.2 ± 9.1 kcal/mol). This suggests that 3-nPBA might be a more potent inhibitor of Mtb LexA autoproteolysis.

**Figure 2:**
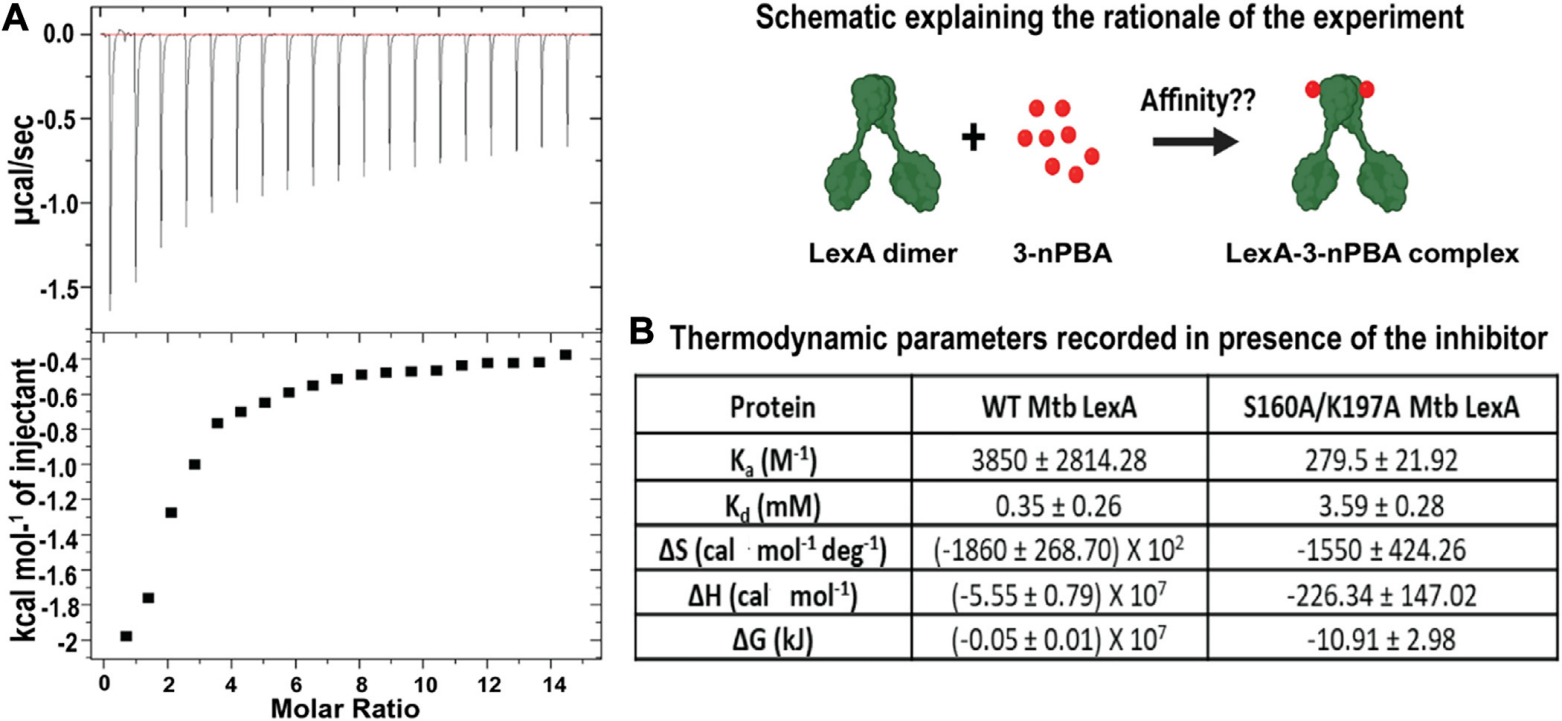
**ITC analysis of Mtb LexA-inhibitor interaction.***A*, binding isotherm of WT Mtb LexA with 3-nPBA.Schematic explaining the rationale behind determining the kinetic parameters of interaction between Mtb LexA and 3-nPBA. *B*, thermodynamic parameters were recorded upon the addition of 3-nPBA.

To experimentally validate these studies, we checked for the direct interaction of the protein with the inhibitor using isothermal titration calorimetry (ITC) and observed that 3-nPBA interacts with Mtb LexA with an affinity of 0.35 ± 0.26 mM (Fig. 2, *A* and *B*). The reaction is thermodynamically favorable and spontaneous, having negative Gibbs free energy. Moreover, to probe whether the catalytic site residues of the protein are involved in inhibitor binding as predicted by docking, we generated mutant(s) of the catalytic site residues using site-directed mutagenesis (SDM) and assessed their effect on inhibitor binding using ITC. Upon mutating both the catalytic site residues which are responsible for its autoproteolytic cleavage (S160A and K197A), we found a 10-fold reduction in affinity for 3-nPBA (Fig. 2*B*). This strongly suggests that mutating the residues responsible for the autoproteolysis of Mtb LexA results in impaired inhibitor binding activity (Fig. S2). Experimental validation using ITC provided a strong indication that the inhibitor could directly interact with Mtb LexA to affect its autoproteolysis, a consequence of which may result in SOS inhibition.

### 3-nPBA stabilizes the structure of Mtb LexA

**Figure 3:**
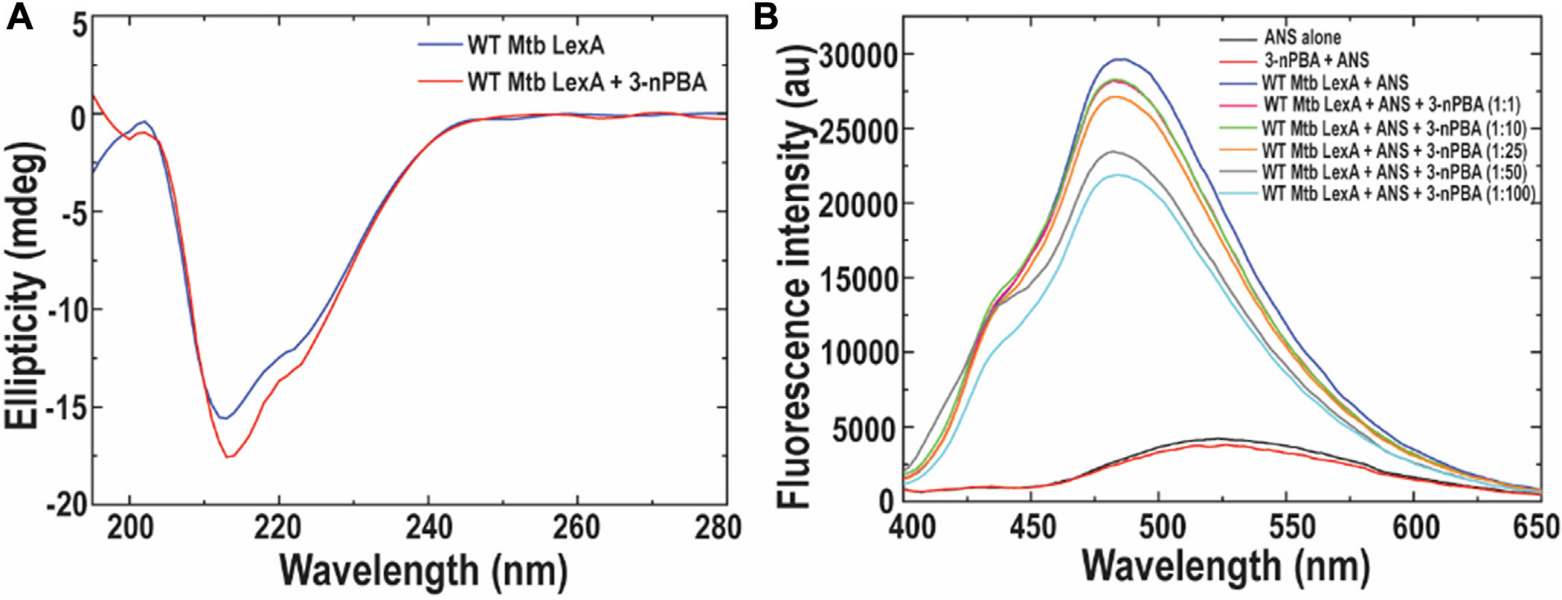
**Characterization of Mtb LexA with potential inhibitor.***A*, secondary structural changes of Mtb LexA with 3-nPBA as determined by CD Spectroscopy. *B*, tertiary structural changes of Mtb LexA upon increasing concentrations of 3-nPBA as determined by extrinsic fluorescence-based studies. Fluorescence intensity is shown in arbitrary units.

To gain more insight into the changes in the secondary and tertiary structure of Mtb LexA with the inhibitor, we performed circular dichroism and extrinsic fluorescence-based studies respectively, wherein we compared the near UV spectra of Mtb LexA alone and with 3-nPBA. Treatment with the inhibitor resulted in increased alpha-helical content of the protein as deduced from the appearance of a more prominent peak at 222 nm of the inhibitor-protein complex as compared to the spectra of the native protein alone (Fig. 3*A*). Moreover, an increase in negative ellipticity is indicative of enhanced protein stability with the inhibitor. For assessment of Mtb LexA tertiary structure changes with varying concentrations of the inhibitor (3-nPBA), fluorescence-based studies using the extrinsic fluorophore, ANS (8-Anilinonaphthalene-1-sulfonic acid), were performed and the obtained spectra showed concentration-dependent quenching in fluorescence (Fig. 3*B*). As ANS is known to bind to the hydrophobic patches of proteins, the elevated fluorescence intensity of ANS indicates increased unfolding of the protein to reveal the hydrophobic patches (39). In this case, the protein possibly gets more folded and stabilized with the inhibitor, resulting in fluorescence quenching.

### 3-nPBA protects Mtb LexA from autoproteolysis without affecting dimerization

**Figure 4:**
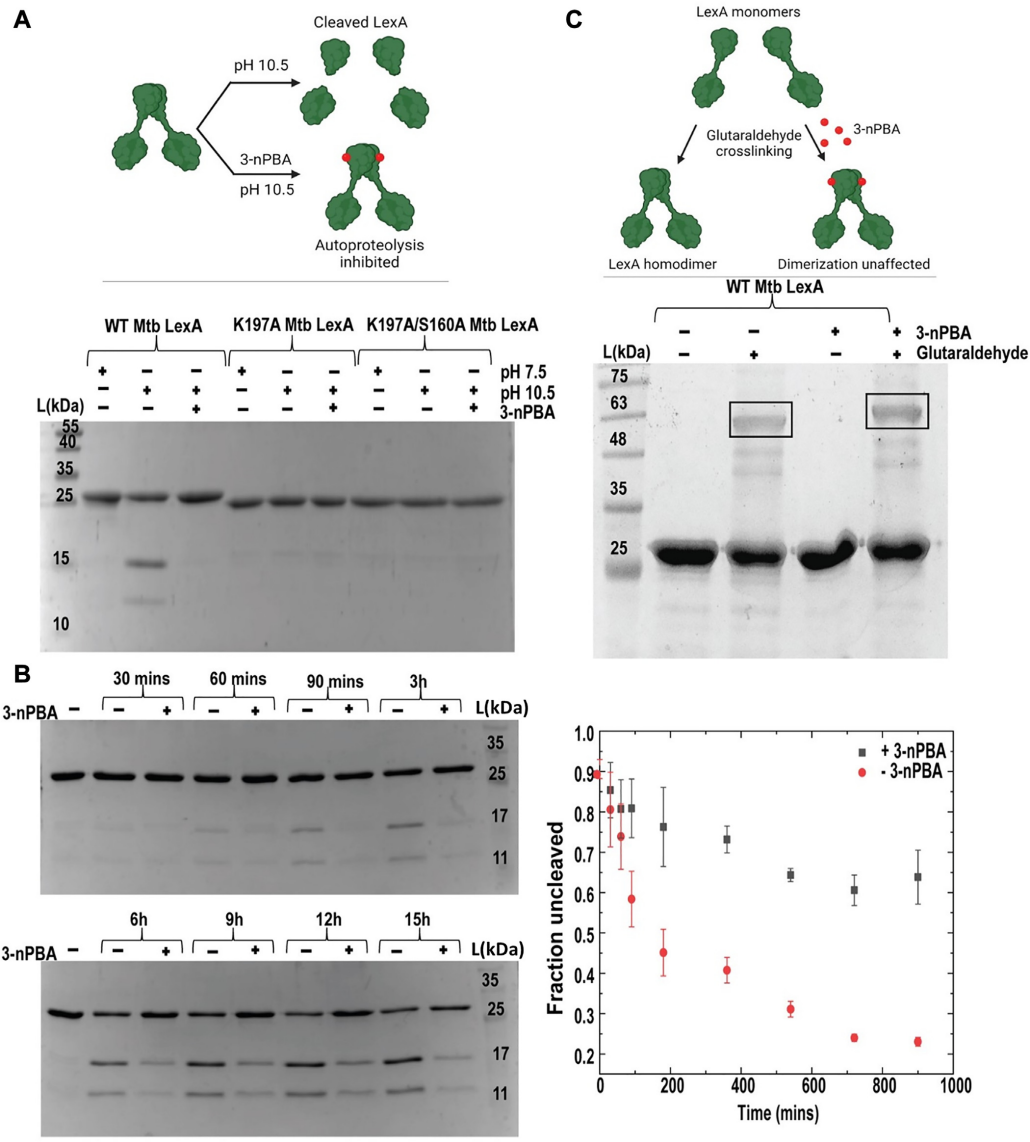
**3-nPBA protects Mtb LexA from autoproteolysis without affecting dimerization.***A*, Autoproteolytic cleavage assay of Mtb LexA and its mutants upon addition of 3-nPBA. *B*, time-dependent cleavage assay of Mtb LexA with and without the inhibitor performed in triplicates quantified in the graph shown on the side. *C*, glutaraldehyde cross-linking assay of Mtb LexA with and without SOS inhibitor, 3-nPBA, reveals no change in the dimerization state of the protein.

Next, we assessed the effect of 3-nPBA on the autoproteolytic activity of Mtb LexA and we found the inhibitor to display a protective effect on the protein, preventing it from undergoing autoproteolysis (Fig. 4*A*). We also assessed the time-dependent inhibition of Mtb LexA autoproteolysis in the presence of 3-nPBA (Fig. 4*B*). Autoproteolysis of LexA occurs at alkaline pH (15, 40). Mutating the residues, K197 and S160, prevented autoproteolysis as observed in Figure 4*A*.

LexA has been reported to bind to its SOS boxes in the nanomolar range, as a dimer (34, 41). We examined whether the inhibitor affected the dimerization property of Mtb LexA using a glutaraldehyde crosslinking assay. We observed that dimerization remained unaffected upon treatment with the inhibitor (Fig. 4*C*).

### SOS inhibitor stabilizes Mtb LexA-DNA complex

**Figure 5:**
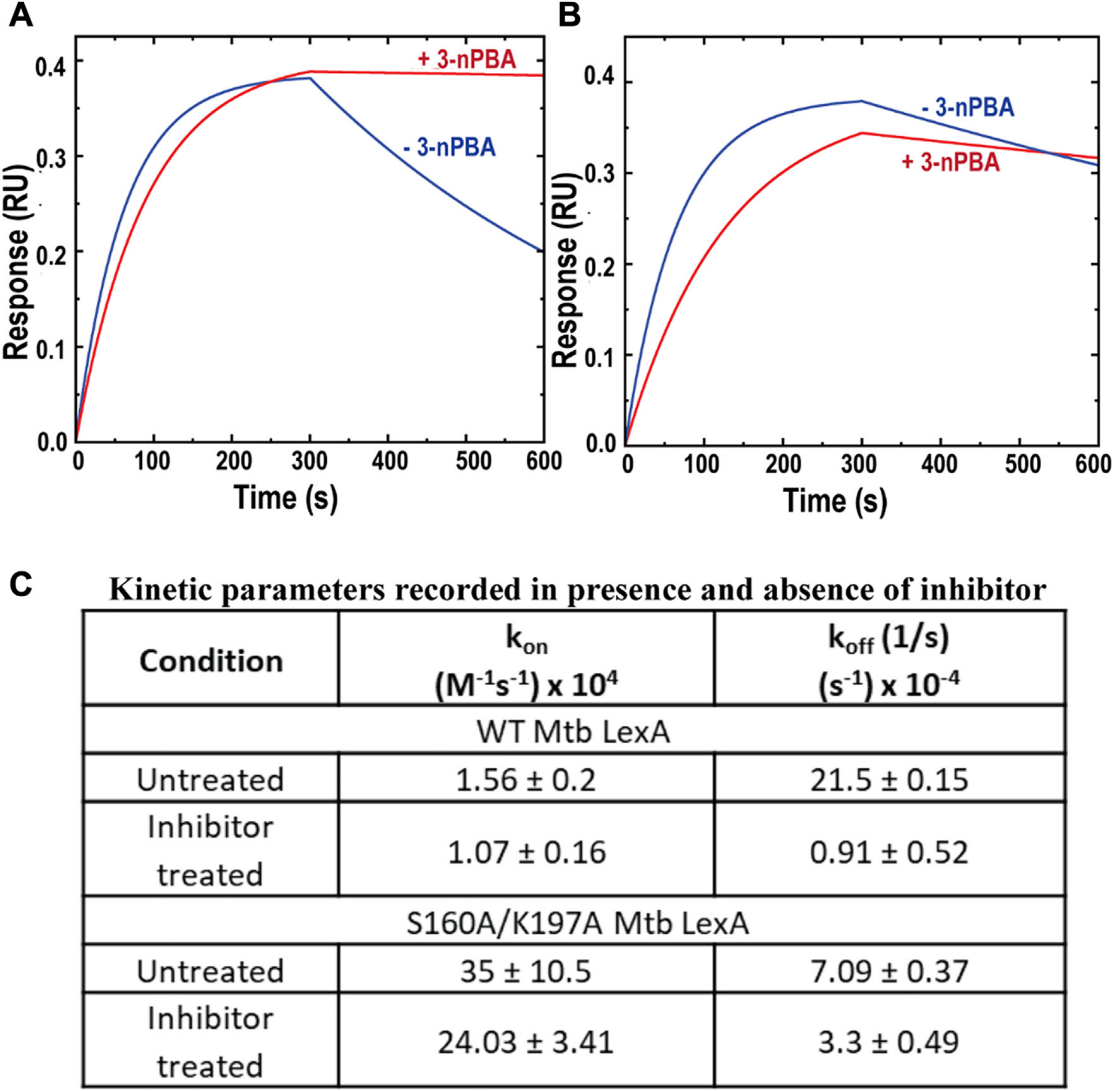
**Dissociation of Mtb LexA assessed with SOS inhibitor, 3-nPBA.***A*, slower dissociation of Mtb LexA from DNA seen in presence of inhibitor as revealed by real-time kinetic studies. *B*, no significant change in the dissociation rate of S160/K197A Mtb LexA from DNA was seen with the inhibitor compared to that observed in (*B*). *C*, Table showing kinetic parameters recorded for the interactions in (*A*) and (*B*).

Although the inhibitor does not impact the process of dimerization, it inhibits the autoproteolytic cleavage of LexA causing the inhibition of SOS response. We hypothesized that the inhibitor might play a role in stabilizing the LexA-DNA complex. To test the hypothesis real-time kinetic studies were performed using biolayer interferometry (BLI) to assess how the inhibitor would affect Mtb LexA-DNA interaction. The DNA used here contained the SOS box of *dnaE2*.The gene *dnaE2* gets expressed upon initiation of the SOS response and is implicated in error-prone mutagenesis (42, 43, 44). Although the association constant (k_on_) of the Mtb LexA-DNA interaction did not alter substantially when compared between the untreated and inhibitor-treated conditions, there was a notable decrease of nearly 23 times in the dissociation constant (k_off_) in the latter case (Fig. 5, *A* and *C*). We observed that in the case of the wild-type protein-DNA interaction, there was an increase in DNA binding affinity by nearly 16 times when treated with 3-nPBA when compared to the untreated condition. Contrarily, in the mutant protein, this difference in the dissociation rate was not significant (Fig. 5, *B* and *C*). Finally, the cumulative affinity of the mutant towards DNA decreased upon treatment with 3-nPBA by 1.5 times when compared to the untreated condition. We observed that the dissociation rate of Mtb LexA from its target DNA sequence decreased with the inhibitor, demonstrating the stabilization of Mtb LexA-DNA interaction by 3-nPBA (Fig. 5, *A* and *C*). To summarize, Mtb LexA binds to 3-nPBA with its catalytic site residues, which prevents its cleavage and also stabilizes its association with DNA. This consequently would prevent the repressor protein from falling off the SOS boxes, thereby maintaining the suppression of SOS-responsive genes.

### Inhibitor-mediated suppression of SOS response

**Figure 6:**
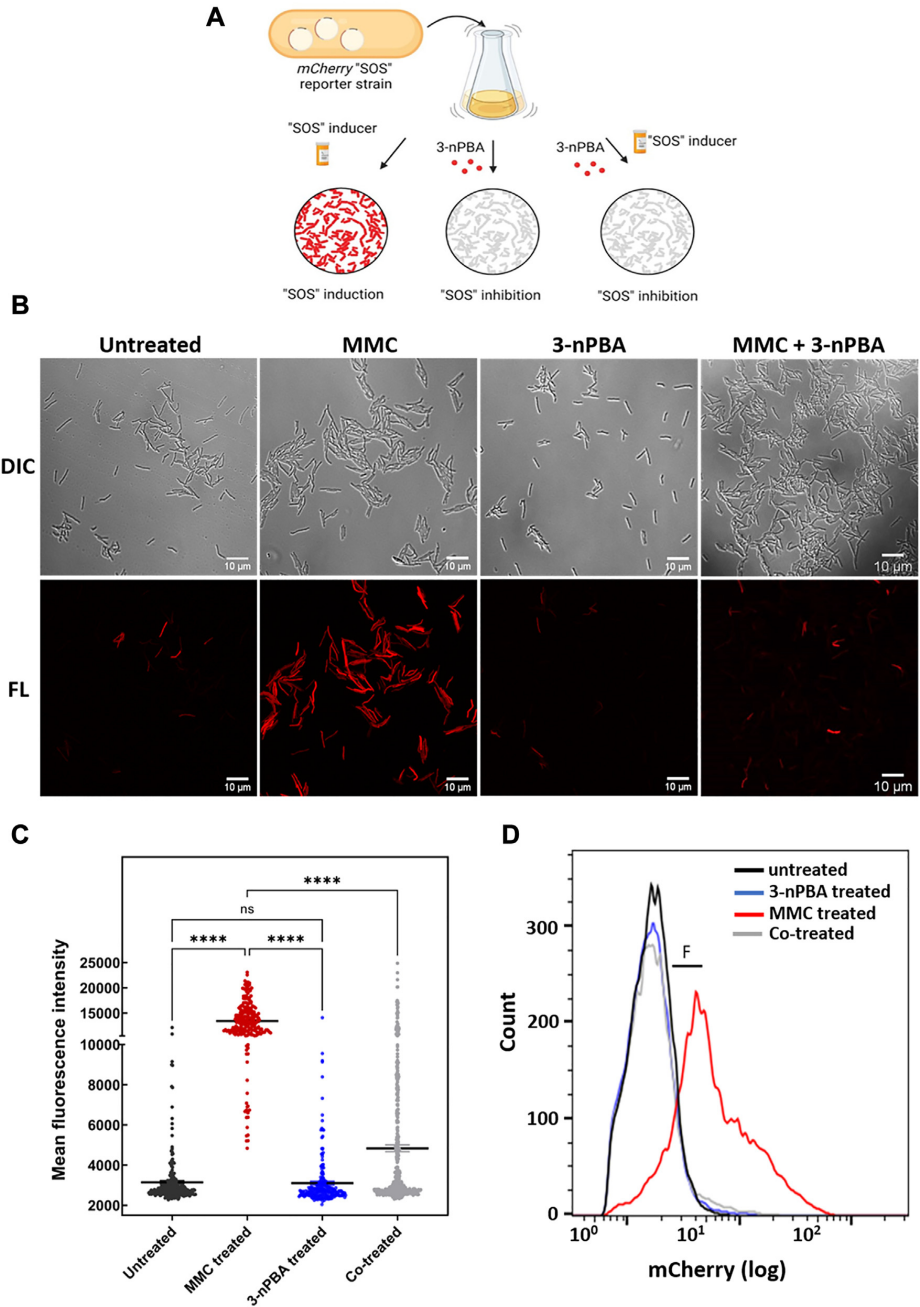
**Assessment of SOS inhibitory activity of the potential inhibitor, 3-nPBA using a fluorescence-based reporter construct.***A*, The strategy used to test the fluorescence-based mycobacterial SOS inducible reporter. *B*, expression of mCherry with a known SOS inducer, mitomycin C can be observed in contrast to the co-treated cells with the potential SOS inhibitor, 3-nPBA, whereby SOS induction gets repressed as observed in the representative confocal microscope images. *C*, quantification of cells under varying treatments. One-way ANOVA was performed (∗∗∗∗ = *p* < 0.0001, ns = non-significant). *D*, representative population histogram of reporter showing increased fluorescence (shown as “F” in the plot) under MMC treated condition as compared to untreated, 3-nPBA treated and co-treated cells.

We generated a fluorescence-based SOS reporter construct containing the promoter region inclusive of a consensus SOS box sequence of *dnaE2* to visualize the SOS response in mycobacteria. We hypothesized that mycobacterial LexA would bind to the SOS box under normal conditions. Upon DNA damage and induction of SOS response, LexA undergoes autoproteolysis and falls off from the SOS box, thereby de-repressing the downstream reporter gene (in this case, *mCherry*) (Fig. 6*A*). Using this SOS reporter construct, we assessed the SOS inhibitory activity of 3-nPBA. As expected, we found SOS activation to be compromised in its presence even at a concentration much lower than the killing concentration of the inhibitor (Fig. 6, *B* and *C*). Mitomycin C (MMC) is a known inducer of the SOS response and it functions by crosslinking DNA and inducing double-strand breaks (45). While SOS-induced samples (treated with MMC) harbored cells expressing *mCherry*, those treated with the inhibitor alone or when co-treated with both the inhibitor and inducer exhibited basal level fluorescence, indicative of sustained SOS inhibition. It is important to mention here that cells exhibit a basal level of SOS response and even in the untreated condition, we can observe some cells expressing *mCherry* in the population as expected (Fig. 6*B*). We also carried out cell sorting of our reporter strain under varying conditions to further validate the results of our microscopy data. Under MMC, stress, we observed a significant increase in *mCherry* fluorescence as compared to the untreated and 3-nPBA treated samples (Fig. 6*D*). A clear reduction in *mCherry* expression could be seen in the co-treated cells as compared to the MMC-treated cells. The cells that showed highly reduced fluorescence upon treatment with the inhibitor at a concentration much lower than the killing concentration of the inhibitor, were still viable as determined from the Resazurin Reduction Assays (REMA) (Fig. S5). To explore this further, we treated a constitutively expressing *mCherry* reporter strain with the inhibitor at the same concentration (lower than the killing concentration of the inhibitor concentration) as used in the case of the fluorescence-based SOS reporter and checked for fluorescence. The treated cells continued to exhibit fluorescence, indicative of their viability (Fig. S3). Hence, we can conclude that treatment with the inhibitor indeed suppressed the SOS response without affecting cell survival. Further, a colorimetric-based assay was also carried out to validate the observation. As expected, we found increased expression of β-galactosidase observed with the SOS inducer, MMC, in contrast to the untreated and 3-nPBA alone treated cells. Cells co-treated with the potential SOS inhibitor, 3-nPBA, showed reduced β-galactosidase activity, indicating suppression of MMC-induced SOS response (Fig. S4). We estimated the antibacterial activity of 3-nPBA against different bacterial strains- *Mycobacterium smegmatis*, the avirulent strain and the virulent strain of Mtb (Mtb H37Ra and Mtb H37Rv, respectively), and against representative Gram-positive and negative organisms. We found the compound to be pan-bactericidal (Table 1, Fig. S5). Moreover, cytotoxicity assessment studies conducted on macrophage cell line RAW264.7 revealed that even at 29 times the MIC of the inhibitor (5212 μM), no observable cytotoxic effect could be observed (Fig. S6). Hence, 3-nPBA can be considered non-cytotoxic for mammalian cells even at higher concentrations.

**Table 1:** MICs against SOS inhibitors and SOS inducer

Bacterial strains	Minimum inhibitory concentration (MIC) of compounds tested		
	3-nPBA (μg/ml)	3-aPBA (μg/ml)	Ciprofloxacin (μg/ml)
*M. smegmatis*	30	625	0.125
*M. tuberculosis* H37Ra	90	1250	0.5
*M. tuberculosis* H37Rv	250	>2000	0.5
*E. coli* BL21 DE3	250	>2000	0.01
*S. aureus*	250	>2000	0.125

### 3-nPBA is anti-mutagenic, curbs expression of SOS regulon genes, and stalls SOS response

**Figure 7:**
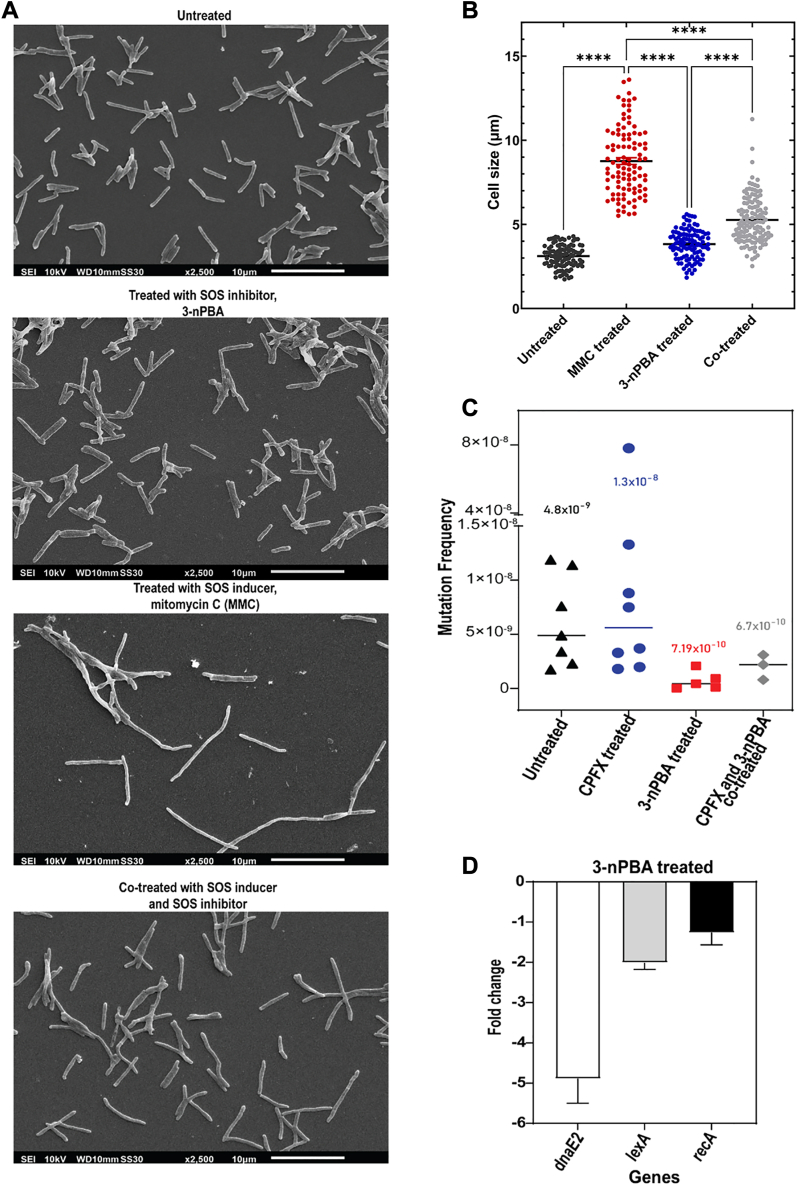
**Deciphering the SOS inhibitory, anti-mutagenic effect of SOS inhibitor 3-nPBA from mutation frequency analysis and differential gene expression studies.***A*, W-SEM images showing variation in cell length upon treatment with SOS activator MMC as compared to the untreated, 3-nPBA, and co-treated samples. *B*, changes in cell size have been plotted by analyzing 100 cells from each group and one-way ANOVA was performed (∗∗∗∗ = *p* < 0.0001, ns = non-significant). *C*, relative analysis of mutation frequency on ciprofloxacin (CPFX) containing plates upon treatment of cells with SOS inducer (CPFX, ciprofloxacin), SOS inhibitor (3-nPBA), and with both ciprofloxacin and 3-nPBA with respect to that of the untreated mycobacterial cells. 9 biological replicates were taken for each condition and values were plotted using GraphPad Prism. *D*, Downregulation of mycobacterial SOS regulon genes upon treatment with 3-nPBA using qRT-PCR. The results shown are from three biological replicates, each with triplicates.

Cell elongation is a hallmark of the activated SOS-induced state of the bacteria (46, 47, 48). In our studies, we identified an appreciable increase in the cell length of *M. smegmatis* when induced with a known SOS inducer like mitomycin C. A counter-effect was induced when cells were co-treated with the SOS inhibitor, 3-nPBA. Cell lengths of untreated and 3-nPBA treated cells were comparable (3.12 ± 0.67 and 3.83 ± 0.87 μm, respectively), while those post-MMC treatment significantly increased in size (8.76 ± 1.99 μm). Co-treatment with the SOS inhibitor rescued the effects of mitomycin C treatment to a significant extent (5.31 ± 1.99 μm) (Fig. 7, *A* and *B*).

Next, to check the efficacy of 3-nPBA on the mutation frequency of antibiotic-treated cultures, we performed mutation frequency tests. Ciprofloxacin was taken as a positive control. Ciprofloxacin is a well-established SOS inducer that results in increased expression of DNA damage-inducible genes forming a part of the SOS regulon whose concerted action brings about mutagenesis (49). Expression levels of the SOS inducible gene *dnaE2,* a primary contributor to bacterial error-prone mutagenesis, remained high in ciprofloxacin-induced persisters even after 30 h of culturing in a medium free of antibiotic (43). To test whether 3-nPBA acts as an anti-mutagenic molecule, we subjected mycobacterial cells to treatment with ciprofloxacin, 3-nPBA, a combination of both ciprofloxacin and 3-nPBA and compared these sets with the untreated control. Next, we recovered the cultures in a growth medium free of antibiotics, while reviving the co-treated samples with the inhibitor and subsequently plating them all on ciprofloxacin-containing plates to assess the mutation frequency rate. We found that mutation frequency decreased by 19 times in 3-nPBA co-treated samples in contrast to the ciprofloxacin-treated samples. Treating with ciprofloxacin alone resulted in 3 times higher mutation frequency compared to the untreated control (Fig. 7*C*). We also attempted to delineate the underlying mechanism by assessing the differential expression of selected SOS regulon genes upon treatment with 3-nPBA. As expected, treatment with the inhibitor resulted in the down-regulation of the genes that are highly controlled by LexA. Differential expression of key SOS regulon genes, *dnaE2, lexA,* showed a nearly 5-fold, and 2-fold reduction, implying that even at the level of transcription, the inhibitor remains effective in stalling mycobacterial SOS response (Fig. 7*D*).

## Discussion

There is an overwhelming need to formulate new strategies to counter mycobacterial multidrug resistance (50). The current antibiotic arsenal needs strengthening by complementing with anti-evolution molecules which can interfere with the bacteria’s ability to acquire drug resistance (51). Since the bacterial SOS response pathway is one of the major drivers of its drug resistance (22, 52, 53), targeting the master regulators controlling this pathway can prove to be promising, and significant efforts in this direction are currently underway. The identification of small molecules that can inhibit the SOS response activation has opened up new avenues in the field of antimicrobial therapy (5). The suppression of mycobacterial SOS response by targeting one of the master regulators, RecA, has been found to slow drug resistance (54). However, since RecA bears mammalian homologs, targeting the other master regulator, LexA is deemed to be more attractive. Currently, very few inhibitors have been identified to target the LexA/RecA axis (55) and the unavailability of Mtb LexA inhibitors prompted us to execute this study.

We conducted our search for an effective inhibitor of Mtb LexA, the repressor master-regulator controlling SOS response. Since boronic acid derivatives were established as prominent inhibitors of LexA autoproteolysis, we hypothesized that modifying the side chains of the boron-containing inhibitors could lead to the identification of efficacious Mtb LexA inhibitors. Through our study, we characterized a boronic acid-containing Mtb LexA inhibitor that proved effective in stalling SOS-induced mutagenesis in mycobacteria. Cell-based screening assays using damage-inducible reporter strain was tested with known mutagen and potential anti-mutagenic inhibitor molecules to check for their activity. Since 3-nPBA was found to be effective in stalling SOS and had a more potent killing activity than another derivative, we chose to proceed with it for subsequent studies. 3-nPBA was found to be potent against multiple bacteria, which establishes its sufficient breadth across species role in stalling SOS response. Moreover, its non-cytotoxic nature also proves to be encouraging for designing further studies.

We found that the mutation frequency decreased by 19 times in 3-nPBA co-treated samples in contrast to the ciprofloxacin-treated samples. Treating with ciprofloxacin alone resulted in a 3 times increase in the mutation frequency compared to the untreated control. These results indicate that the SOS inhibitor is indeed anti-mutagenic. Studying the gene expression patterns of mycobacterial SOS regulon genes upon treating with the inhibitor further validated our findings as they were found to be down-regulated in its presence, thereby suppressing SOS-mediated mutagenesis.

Through covalent docking studies, we found the catalytic side residues (S160 and K197) of Mtb LexA to be possibly involved in interacting with 3-nPBA. We generated the catalytic mutant(s) of Mtb LexA, assessed the binding to 3-nPBA, and weighed it against with that of the wild-type protein. The binding studies using ITC revealed compromised inhibitor binding properties of the double mutant. The covalent bond formed between the inhibitor and the protein ensures a stable interaction, and this satisfies the criteria to serve as a promising inhibitor molecule (56).

Further, we performed biochemical and biophysical studies to elucidate the effect of the inhibitor on Mtb LexA. Using autoproteolysis assays to compare the stability of the protein with or without the inhibitor revealed that 3-nPBA has a protective effect on Mtb LexA, preventing its cleavage. We observed stabilizing secondary structural changes of the protein with an inhibitor using circular dichroism studies. Concentration-dependent change in the tertiary structure of the protein with increasing concentrations of the inhibitor was observed using ANS-based extrinsic fluorescence assays. The inhibitor did not affect the dimerizing ability of Mtb LexA, which is a prerequisite for its DNA binding property. We found that 3-nPBA slowed down the rate of dissociation of Mtb LexA from the DNA significantly, through real-time kinetic studies. This indicates that the inhibitor disallows Mtb LexA from falling off the DNA, prevents its autoproteolysis, and stabilizes the Mtb LexA-DNA interaction, thereby repressing SOS activation from taking place.

All the above-mentioned studies taken together provide us with a detailed mechanistic understanding of how this first-of-its-kind mycobacterial SOS inhibitor may help prevent the bacteria from gaining AMR by stalling its SOS response machinery. To date, we did not have anti-mutagenic inhibitors that could target the SOS response axis of mycobacteria. Such inhibitor molecules hold promise in adjuvant therapy to accentuate the activity of existing drug regimens. This study lays the platform for developing an anti-mutagenic SOS inhibitor screening platform that may help target not just *M. tuberculosis,* but also other pathogenic Gram-negative and Gram-positive bacteria, which is much needed in this era of expanding antimicrobial resistance.

## Experimental procedures

### Reagents, plasmids, and strains

Bacterial plasmids and strains used in this study are mentioned in Table S2 of Supporting information. Primer sequences and constructs generated in the study are listed in Table S3 of Supporting information. All the chemicals, reagents, and media utilized were acquired from Hi-Media, Sigma Aldrich, SRL, and Difco, and enzymes were secured from New England Biolabs, Genei, and Promega. 50 mg/ml stocks of the inhibitors (3-nPBA and 3-aPBA) were prepared in 40% DMSO.

### Cell-based reporter assays

Mycobacterial cells bearing the SOS inducible reporter were grown up to O.D_600_ 0.4 and divided into different tubes containing 40 ng/ml of SOS-inducing agent such as mitomycin C, or the inhibitor, 3-nPBA, at one-fourth of its MIC (7.5 μg/ml), or in combination with both the SOS inducer and inhibitor, and grown for 4 h before analysis. Cells washed with 1X PBS were fixed with 3% paraformaldehyde (PFA), and mounted on agar padding before imaging. Samples were observed under 63× magnification of the confocal microscope. An excitation wavelength of 565 nm and an emission filter of 610 nm was taken for observing mCherry-expressing cells. The mean fluorescence of cells under the above-mentioned conditions was analyzed using flow cytometry (Partec CyFlow).

### W-SEM analysis

1 ml of untreated and treated cells were pelleted and washed two times with 1X PBS. Subsequently, the cells were fixed on coverslips with 2.5% (v/v) glutaraldehyde by incubating for 45 min at room temperature. Dehydration of the cells was performed by washing with a graded series of ethanol (30–100%). Finally, the coverslips were mounted on an aluminum stub using two-sided carbon tape. The samples were dried overnight in a desiccator. Samples were gold-coated the following day. Images were captured at 10 kV using a WD detector under a scanning electron microscope.

### Mutation frequency analysis

The protocol followed as per Salini *et al.*, 2022 (43). Additionally, modifications suitable for the experiment were done. The concentration of 3-nPBA tested was half of its MIC concentration against *M. smegmatis* i.e., 15 μg/ml (89.8 μM). The concentration of ciprofloxacin tested was 7 times less than the molar concentration of the inhibitor used, that is, 12.8 μM. Incubation was done for 6 days post which plating was carried out. For viability testing, 10^5^ and 10^7^ dilutions of saturated cultures were plated while the remaining culture was plated as mentioned in the protocol on antibiotic-containing plates.

### RNA isolation, DNase treatment, cDNA conversion, and qRT PCR

For this, the standard procedure as given in (57) with few modifications was followed. As a modification to the mentioned protocol, 0.5 mm Zirconia beads were used for lysis after the addition of Trizol and Chloroform for RNA isolation. DNase treatment using DNase I (Promega) was performed according to the manufacturer’s instructions. 1 μg of RNA from each sample was converted to cDNA following instructions of Promega. qPCR was performed using SyBr green. 65 °C was chosen as the annealing temperature. *rpoB* was used as housekeeping control and as a reference.

### Covalent docking

Details of how protein and ligands were prepared for docking are mentioned in the Supporting information.

### Over-expression and purification of proteins

Mtb LexA construct generated in our previous study was used (34). Mutants were generated by the non-overlapping site-directed mutagenesis. The primer sequences are mentioned in Table S3 of Supporting information. Sequencing was done for the verification all the constructs. The expression of the wild-type and the mutants were done in *E. coli* BL21(DE3) cells following the published protocol (34).

### Isothermal titration calorimetry

The recombinant proteins were titrated against the ligand in Microcal ITC 200 (GE). 20 injections, each of 2 μl, were made at 150 s intervals, at 25 °C. The heat of the reaction in each injection (micro calories per second) was calculated by the integration of the peak areas. The concentration of protein has been calculated taking into consideration its dimeric form in solution. 25 mM phosphate buffer, pH 7.5 was used.

### Circular dichroism

Circular dichroism experiments were accomplished using the Jasco J-815 spectropolarimeter according to our previously published protocol (34). 5 μM of protein in buffer A (50 mM NaCl, 10 mM HEPES (pH 7.5)), and 100X molar concentration of the inhibitor were subjected to evaluation. The data shown are the mean of three distinct runs after mitigating for the buffer baseline. Origin 8.1 software was used for plotting the recorded spectra.

### Extrinsic fluorescence

Extrinsic fluorescence-based studies were performed according to our previously published protocol (34). 5 μM of the protein in buffer A, was incubated with varying concentrations of the inhibitor (3-nPBA) for 30 min at 37 °C. Baseline corrections were done for all measurements (fluorescence intensity of buffer, ANS intrinsic fluorescence, and inhibitor).

### Autoproteolysis cleavage assay

Autoproteolytic cleavage of Mtb LexA and its variants were induced using 100 mM CAPS, 300 mM NaCl (pH 10.5) and by incubating the proteins at 37 °C for 6h, if not mentioned otherwise. 5 μM of each protein was pre-incubated for 3 h with the inhibitor (1:100 ratio) and then subjected to autoproteolysis. For time-dependent studies, incubation of protein with inhibitor was carried out for varying time periods and samples were collected for analysis. Samples in all cases were run on 15% SDS-PAGE. The images were captured using Bio-rad Gel Doc EZ imager and quantified using Image J.

### Cross-linking reactions

The cross-linking experiments were conducted according to the already standardized protocol (34) either with or without the inhibitor (3-nPBA). 1:100 was taken as the ratio of protein to inhibitor. 25 mM DTT was used to stop the reactions. Samples were prepared and separated on 12% SDS-PAGE.

### Biolayer interferometry (BLI)

The interaction studies between Mtb LexA with biotinylated ds 44mer of *dnaE2* SOS box (sequence itemized in Table S3 of Supporting information) with or without the inhibitor, 3-nPBA in a 1:10 ratio of protein: inhibitor was performed using biolayer interferometry according to a previously published protocol (34). After reference data subtraction, a 1:1 binding model was used for fitting and plotting the data.

## Data availability

The authors confirm that the data supporting this study are available within the article and/or its Supporting information, or can be made available upon reasonable request to the first author, Chitral Chatterjee (email id: chitralchatterjee2@gmail.com).

## Supporting information

This article contains supporting information (15, 40, 58, 59, 60, 61, 62, 63, 64, 65, 66).

## Conflict of interest

The authors declare no conflicts of interest with the contents of the article.
