# Supplementary material for: Anti-mutagenic agent targeting LexA to combat antimicrobial resistance in mycobacteria
Source: J Biol Chem. 2024 Aug 8;300(9):107650. doi: 10.1016/j.jbc.2024.107650 (PMC11408154; doi:10.1016/j.jbc.2024.107650)
Supplement: Supporting Figures and Tables [file mmc1.docx]

**Supporting information for**

**Anti-mutagenic agent targeting LexA to combat antimicrobial resistance in mycobacteria**

**Chitral Chatterjee^1^, Gokul Raj Mohan^1^, Hariharan V.C^1^, Bhumika Biswas^1^, Vidya Sundaram^2^,**

**Ashutosh Srivastava^2^ and Saravanan Matheshwaran^1,3,4*^**

^1^Department of Biological Sciences and Bioengineering; Indian Institute of Technology Kanpur, Kanpur-208016, UP, India

^2^Department of Biological Sciences and Engineering, Indian Institute of Technology, Gandhinagar-382355, Gujarat, India

^3^Centre for Environmental Sciences and Engineering, Indian Institute of Technology Kanpur, Kanpur-208016, UP, India

^4^Mehta Family Centre for Engineering in Medicine, Indian Institute of Technology, Kanpur-208016, UP, India

^5^Kotak School of Sustainability, Indian Institute of Technology, Kanpur-208016, UP, India

^*^**Corresponding author:** Saravanan Matheshwaran

**Email:** [saran@iitk.ac.in](mailto:saran@iitk.ac.in)

**Experimental Procedures:**

**Three-dimensional structure of C-terminal domain (CTD) of Mtb LexA: selection and preparation**

At the time of investigation, only a single study on the X-ray structure of the Mtb LexA C-terminal domain (CTD) (1) had been done and the corresponding data was deposited in the Protein Data Bank (PDB). The crystal structure of the CTD has four forms; forms I (PDB ID - 6A2Q) and II (PDB ID - 6A2R) are both unmutated with one and six monomers in the asymmetric units, respectively. The crystal structure with PDB ID 6A2Q with the monomeric CTD was selected for further investigations. The CTD structure was generated using the Protein Prep wizard (2). The protein preparation step included the addition of missing residues and hydrogens, removal of water molecules, optimization of hydrogen bonds, and energy minimization with convergence to a maximum RMSD of 0.3 Å. Although the crystal structure of CTD with a co-crystallized inhibitor is yet to be determined, a probable binding pocket based on the comparative analysis of the CTD of *E. coli* and *P. aeruginosa* has been suggested (3)*.* The immediate neighborhood of the catalytic site residues forms a pocket comprising residue stretches
154-161 and 193-197 that could be considered as a target for the generation of inhibitors; stretch
154-161 is substantially the same in LexA from Mtb, *E. coli* and *P. aeruginosa,* and stretch
193–197 is extremely conserved in *E. coli* and *P. aeruginosa*. Accordingly, the energy-minimized protein structure was then processed in the Glide module (4–6) to generate grids with the centroid defined by residue stretches 154-161 and 193-197.

**Ligand Preparation**

LigPrep with Epik (7) was employed to generate the three-dimensional structures of the ligands mentioned in **Table S1**. The generated structures were then optimized at pH 7.0 ± 0.5. The processed protein structures with the grid and the ligands were subjected to two docking procedures; conventional molecular docking in the Glide module and covalent docking using the CovDock module (8) of Schrodinger, both at extra-precision (XP) mode (4).

**Covalent Docking**

For covalent docking involving boronic acid derivatives, the reaction type and reactive residue on the receptor were identified as boronic acid addition and the catalytic S160, respectively. Further, the CovDock module of Schrodinger was used to dock the molecules to the catalytic site of
Mtb LexA. The CovDock affinity score and MMGBSA score were reported for all the molecules
(**Table S1**).

**Molecular Dynamics Simulations**

To analyze stability of the protein-ligand complexes, molecular dynamics (MD) simulations were performed for chosen complexes using Desmond (9). Each of the selected protein-ligand complexes was simulated in a truncated octahedron box solvated with explicit TIP3P water molecules. OPLS3e force field (10) was utilized to simulate the inhibitor and protein molecules. The default energy minimization and equilibration settings of Desmond were applied to the simulation system. The equilibrated systems were used for the final production run of 10 ns in the NPT ensemble at 1 atm pressure, 310 K. The stability of the protein-ligand interactions was analyzed by utillising the “simulation integration diagram” and “simulation quality analysis” tools of Desmond.

**Growth conditions**

*M. smegmatis* cultures were grown in 7H9 media with 0.2 % Tween-80 (v/v), 0.5 % glycerol. For growing Mtb H37Ra and Mtb H37Rv, OADC was used as a supplement in 7H9. 7H11 agar with 0.5 % glycerol was added for solid media. 25 μg/ml kanamycin and 50 μg/ml hygromycin was used as and when required. Strains were cultured by incubating at 37°C. LB was utilized to culture *E. coli* and *S. aureus* and while performing resazurin reduction assays, secondary cultures were grown in MH broth.

**β-galactosidase assay**

For generating a colorimetric based reporter construct using the promoterless vector (pSD5B), SOS box containing sequence of a DNA damage inducible gene (in this case, *rv1378c*) was cloned between XbaI (forward) and SphI (reverse) sites. β-galactosidase expression was monitored in response to SOS activation and inhibition. To test its potential, the reporter construct was electroporated into *M. smegmatis* and the electroporated native vector served as a control. Untreated and treated cells were grown to late log phase and harvested for
β-galactosidase assay. mitomycin C (MMC) was used for activating SOS response. The assay was performed in accordance with (11). Comparisons were made based on colorimetric intensity, which is indicative of damage induction.

**MIC Determination using Resazurin Reduction Assay**

For this, standard procedure was followed for *M. smegmatis* (12). After setting up the plates, they were incubated at 37ºC for 40 hours at 100 rpm post which, 0.2 mg/ml final concentration of resazurin was added with further incubation for 6 hours after which images were taken. For testing in Mtb H37Ra, the following protocol was followed. Briefly, 200 µl MilliQ was added to the perimeter wells of a 96-well plate. Cultures of Mtb H37Ra were grown up to O.D_600_ 0.6 in replicates and were diluted to 0.02. 100 µl of growth media was added in the required wells. Drugs were added from individual stocks to achieve the required final concentrations in the wells. 100 µl of culture was pipetted to the wells to make up to 200 µl. Incubation was done at 37°C for 7 days. 30 µl of 0.01 % resazurin was added (final concentration 0.0015 %), incubated for 24 hours, and imaged. For testing in *S. aureus*, replicates of cultures were grown up to
O.D_600_ 0.6 and diluted to 0.0008. 80 µl of culture was added with 20 µl of drugs from a 5 X higher stock. Plates were incubated for 24 hours at 37°C without shaking. 30 µl of resazurin was finally added to a concentration of 0.02 % and incubated for 2 hours at 37°C without shaking, post which images were taken. The protocol remained the same when followed for
*E. coli* BL21DE3, with the only change being that the plates were incubated for 16 hours instead of for 24 hours as done for *S. aureus.* In all cases, ciprofloxacin was used as a positive control. Other relevant controls were taken as required. All experiments were performed in biological replicates as well as in technical replicates.

**Cytotoxicity assessment**

The percentage of cell viability was assessed with Resazurin Reduction Assay in RAW 264.7 cell line by treating with the inhibitor. 10,000 cells/well were seeded in a 96-well plate. After 24 h varying concentrations of 3-nPBA were applied. Treated cells were allowed to grow for another 48 h. Then, resazurin was added to each well (30 μl from a 0.01 % stock for a 200 μl reaction) and incubated for another 24 hours before taking readings. The percentage of cell viability was determined.


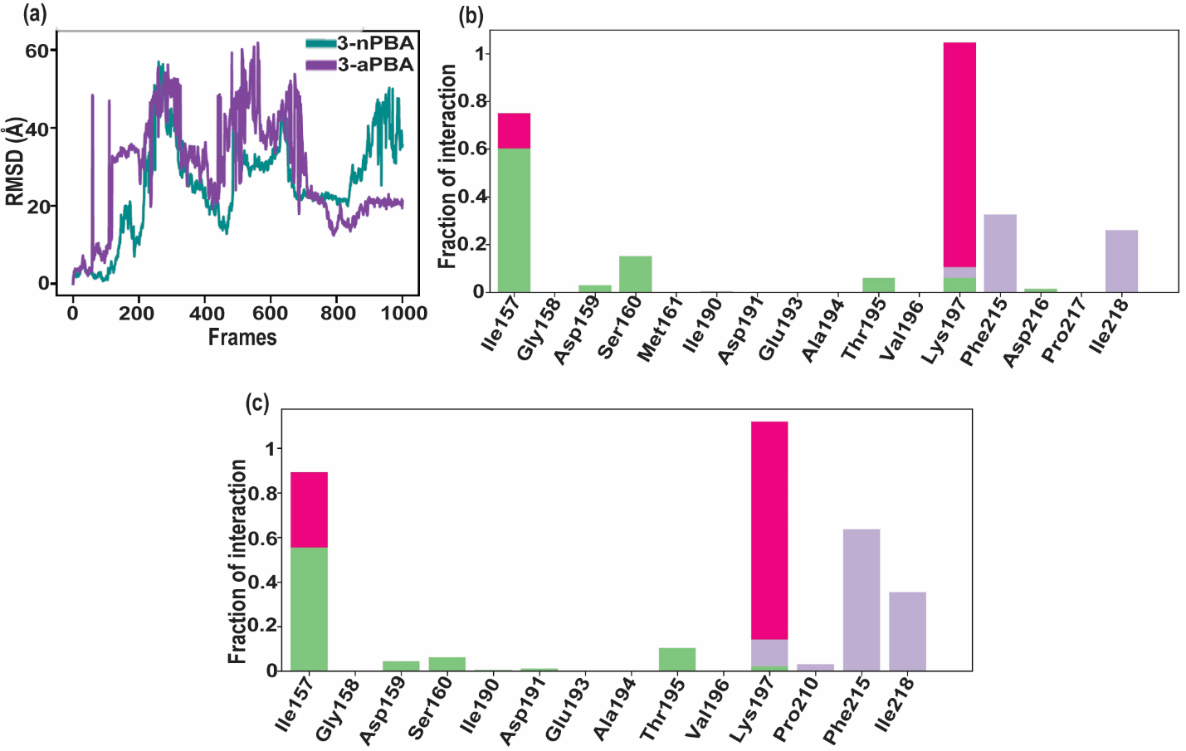


**Figure S1 (a) Root Mean Square Deviation of 3-nPBA and 3-aPBA during 10 ns (1000 Frames) MD simulations of
glide-docked complexes. (b) Ligand atom interactions of 3-nPBA with Mtb LexA during 10ns run of covalently docked complex. shown as stacked bar charts. (c) Ligand atom interactions of 3-aPBA with Mtb LexA during 10ns run of covalently docked complex. shown as stacked bar charts. Hydrogen bonds (green color), hydrophobic interactions (purple) and ionic interactions (magenta color) were shown.**


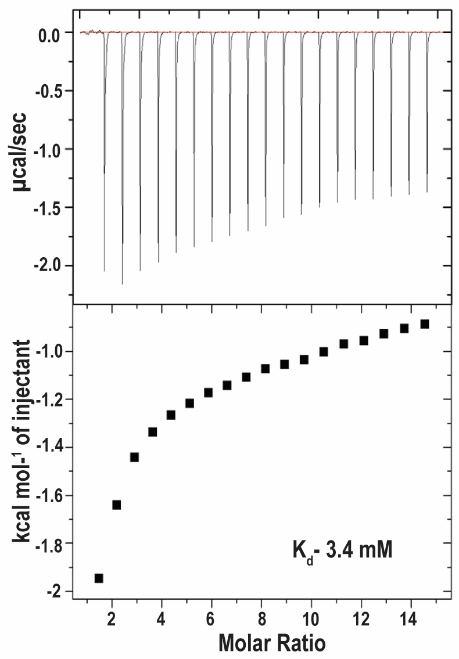


**Figure S2. Binding isotherm of S160A/K197A Mtb LexA with 3-nPBA.**


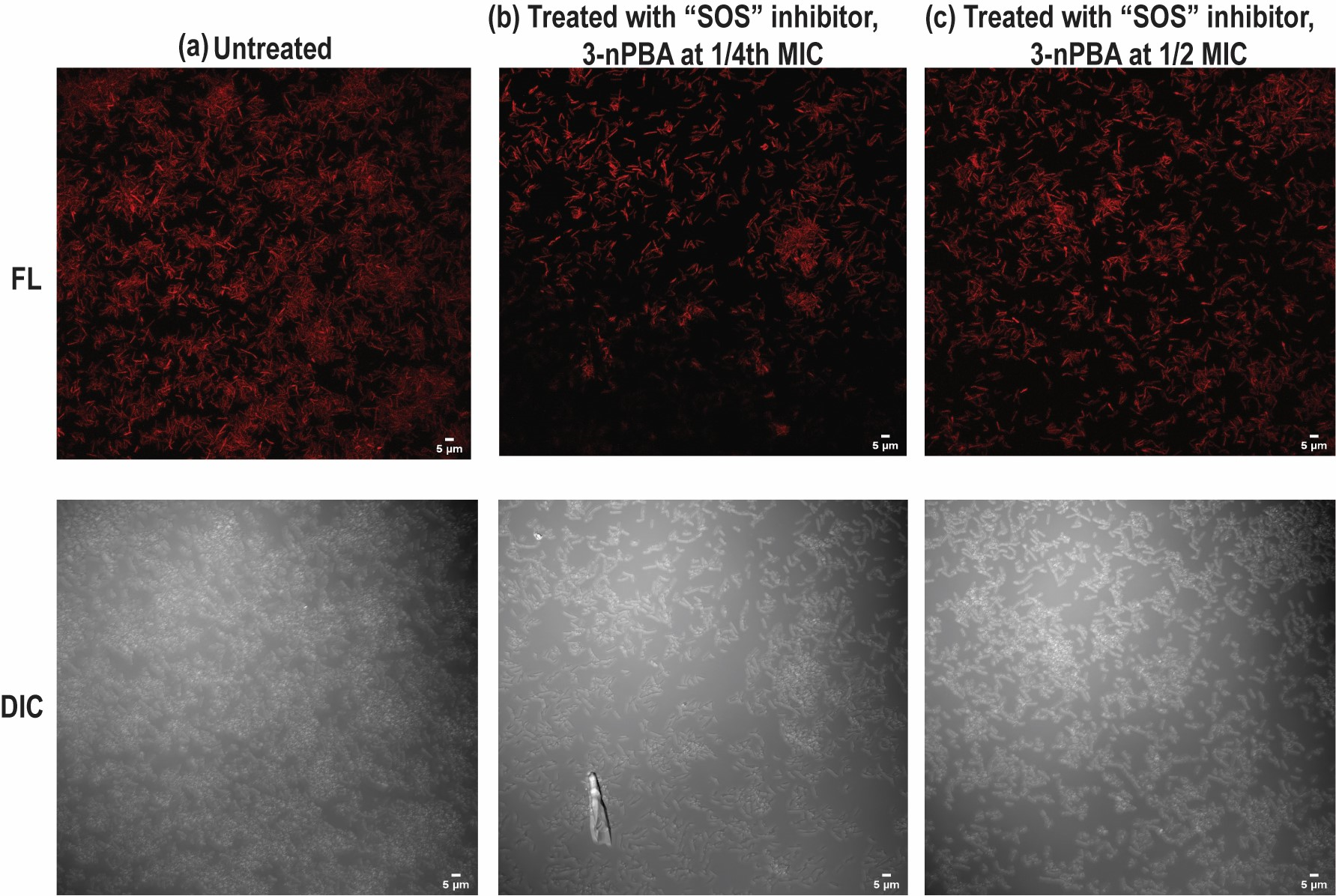


**Figure S3. Treatment with SOS inhibitor does not affect viability of cells. *M. smegmatis* cells transformed with pMV262~*mCherry* whereby *mCherry* gets constitutively expressed was used for this study. Treatment with the SOS inhibitor at both (b) 1/4th and (c) 1/2 of its minimum inhibitory concentration (MIC) is not seen to affect cell viability when compared to the (a) untreated control as the cells continue to exhibit fluorescence, which is an indication of their viable state.**


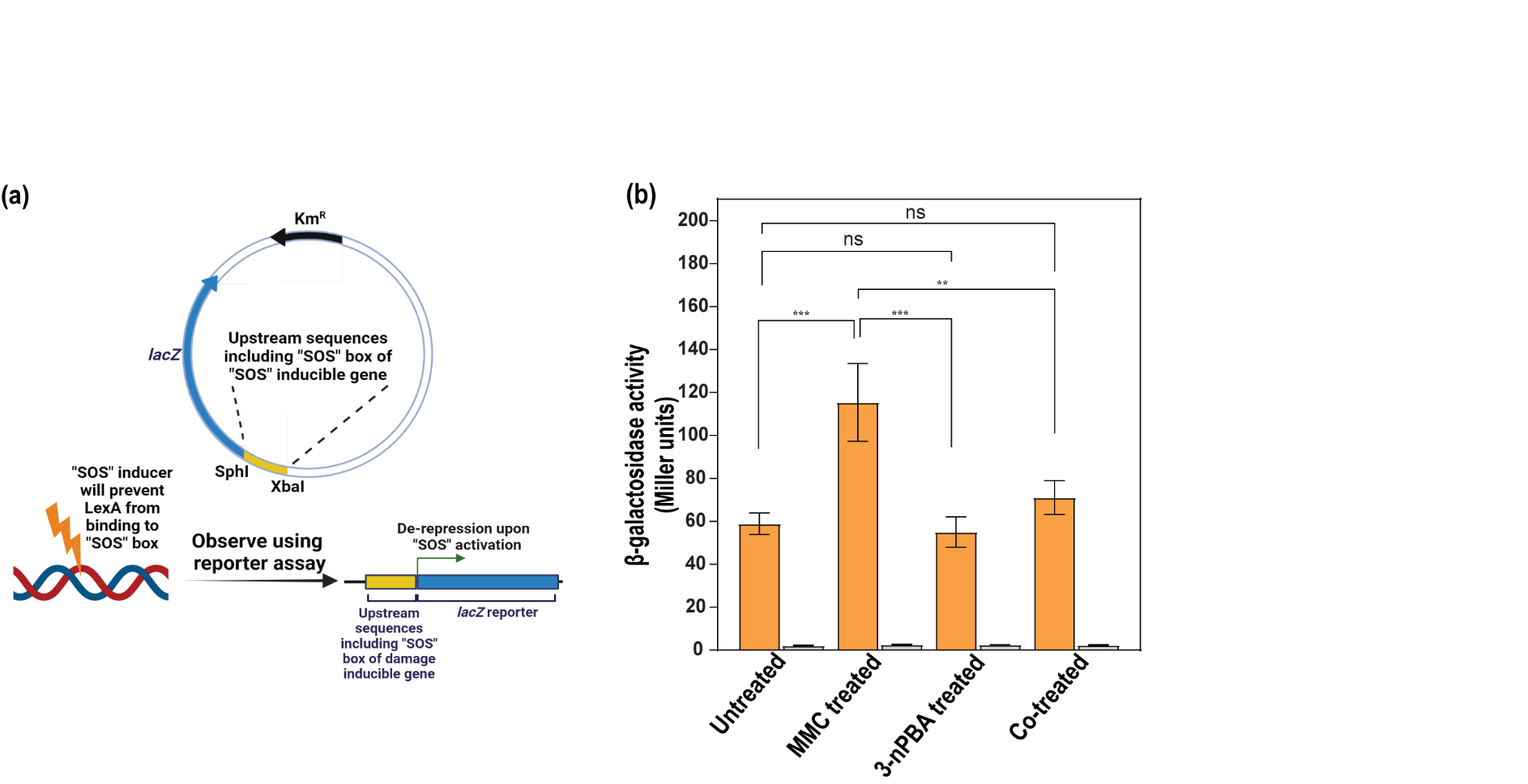


**Figure S4. Assessment of SOS inhibitory activity of the potential inhibitor, 3-nPBA using colorimetric-based reporter assay. (a) Strategy used to construct the colorimetric-based mycobacterial SOS inducible reporter.
(b****) Higher expression of β-galactosidase observed with a known SOS inducer, mitomycin C, in contrast to the untreated and 3-nPBA alone treated cells. Here, orange bars represent test samples while the gray bars indicate vector controls treated in the same manner. Co-treating cells with the SOS inhibitor, 3-nPBA, significantly reduced SOS induction as shown by reduced β-galactosidase activity. The data is represented from 3 replicates. One-way ANOVA was performed (**=p<0.01, ***=p<0.001, ns=non-significant).**


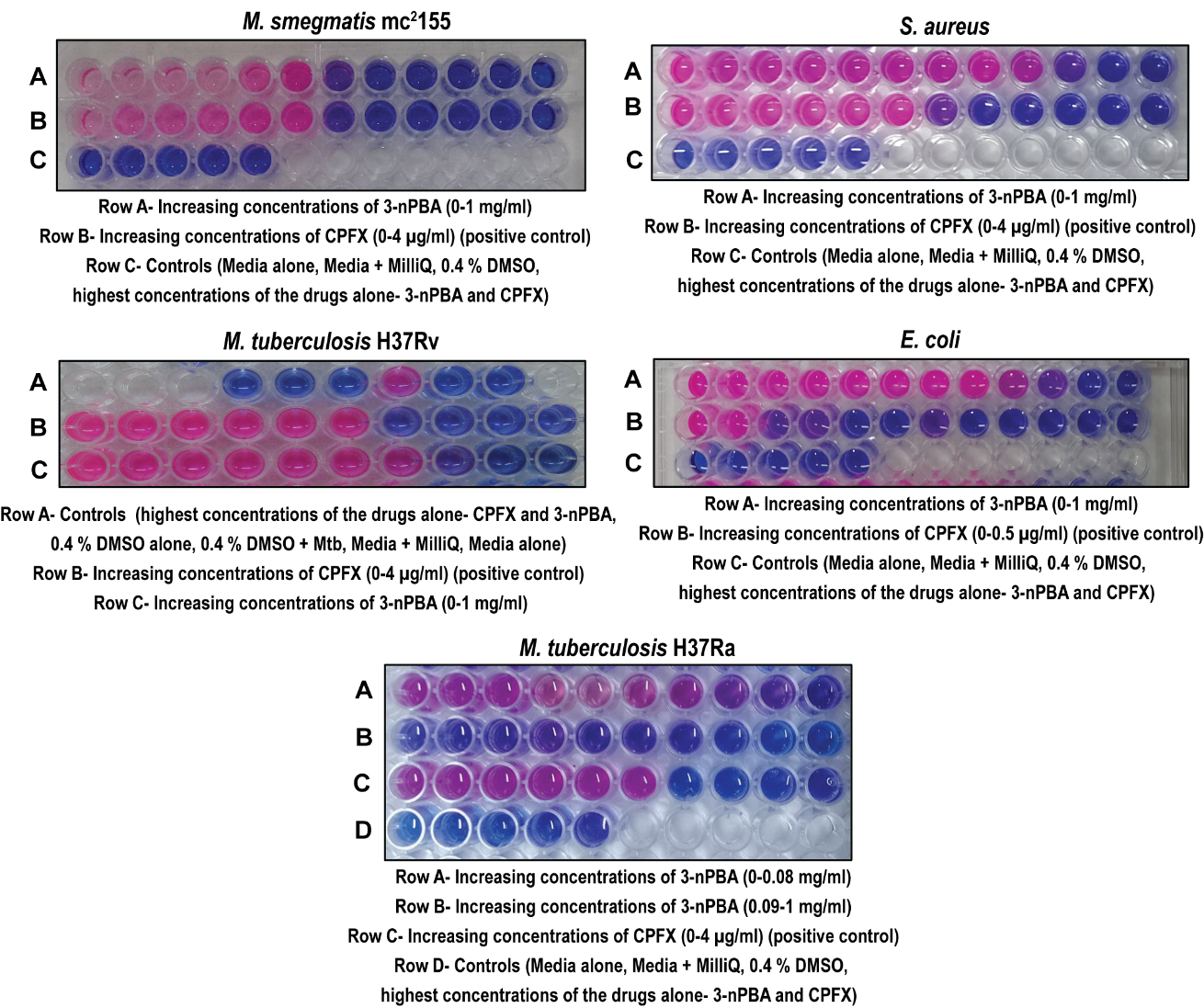


**Figure S5. Determination of MIC of SOS inhibitors (3-aPBA and 3-nPBA) and ciprofloxacin (SOS inducer) against mycobacterial species and representative Gram-positive and Gram-negative organisms.**


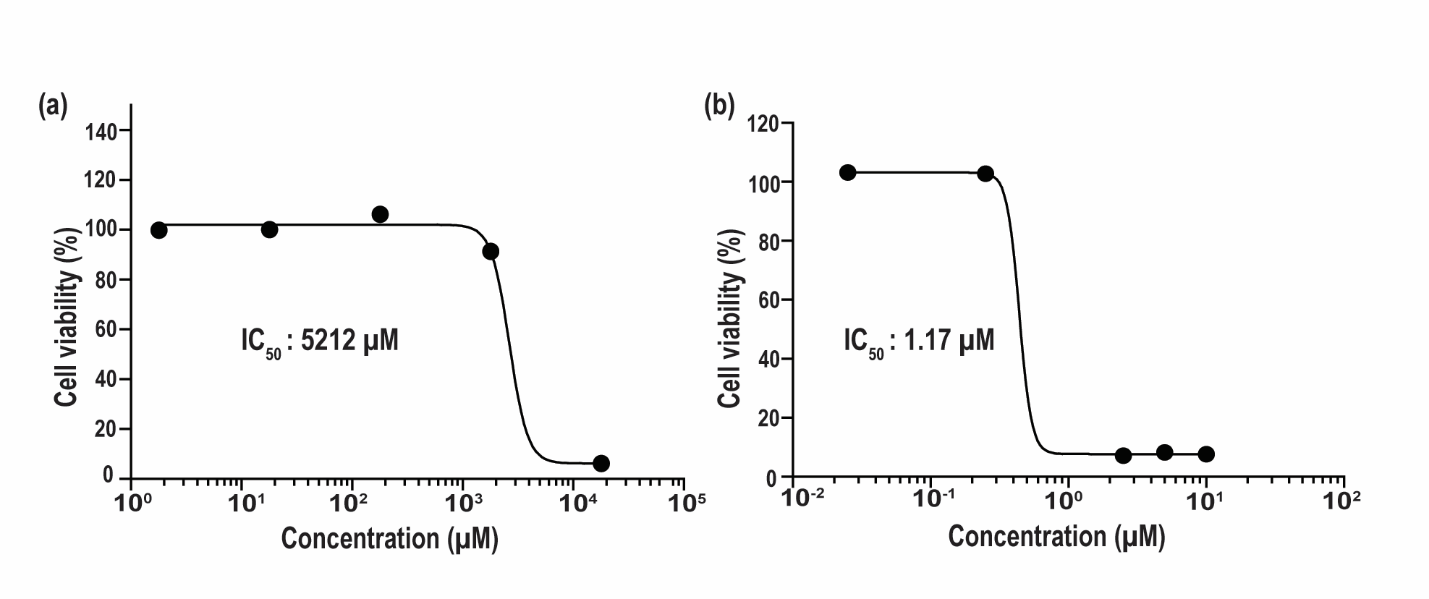
**Figure S6. Treatment with SOS inhibitor does not affect viability of RAW 264.7 cells (a). Doxorubicin was taken as positive control (b).**

**Table S1: Compounds screened in this study**

| **Compound** | **Chemical structure** | **MMGBSA**  **dG Bind​** | **cdock affinity** | **Glide Score** |
| --- | --- | --- | --- | --- |
| Phenylboronic acid |  | -2.78 | -3.281 | -2.446 |
| 3-aminophenylboronic acid |  | -1.93 | -2.861 | -3.341 |
| **3-nitrophenylboronic acid** |  | **-4.96** | **-3.15** | **-3.267** |
| Benzene 1,4 di-boronic acid |  | -2.99 | -2.446 | -4.274 |

| 4-pyridylboronic acid |  | 2.9 | -3.466 | -3.512 |
| --- | --- | --- | --- | --- |
| 4-formylphenylboronic acid |  | 11.87 | -3.76 | -3.501 |
| 3-hydroxyphenylboronic acid |  | 1.53 | -3.391 | -3.59 |
| 3-carboxyphenylboronic acid |  | 1.17 | -3.285 | -3.128 |
| [3-(2-carboxyvinyl)phenyl]  boronic acid |  | 1.53 | -2.642 | -3.819 |
| Vaborbactam |  | Not determined | -3.713 | -3.463 |
| Bortezomib |  | 13.95 | -2.874 | -4.107 |
| Ixazomib |  | 2.87 | -1.487 | -4.949 |

# Table S2: Bacterial strains and plasmids

| **Name** | **Characteristics** | **Source** |
| --- | --- | --- |
| **Bacterial Strains** | | |
| *E. coli* DH5α | Wild-type *E. coli* K12 strain. | Laboratory stock |
| *E. coli* BL21(DE3) | *E. coli* strain used routinely for recombinant protein expression and purification. | Laboratory stock |
| *M. smegmatis* mc^2^155 | High-efficiency transformation strain of  *M. smegmatis.* | Laboratory stock |
| pMV262~*mCherry* mc^2^155 strain | *M. smegmatis* mc^2^155 transformed with pMV262~*mCherry* replicative plasmid and is resistant to kanamycin. | This study |
| *P_dnaE2_*~*mCherry* mc^2^155 reporter | *M. smegmatis* mc^2^155 transformed with 150bp upstream cloned of *M. tuberculosis* *dnaE2* gene containing its SOS box region upstream of *mCherry* reporter plasmid and is resistant to kanamycin. | This study |
| pSD5B~*lacZ* mc^2^155 strain | *M. smegmatis* mc^2^155 transformed with pSD5B~*lacZ* replicative plasmid and is resistant to kanamycin. | This study |
| *P_rv1378c_*~*lacZ* mc^2^155 reporter | *M. smegmatis* mc^2^155 transformed with 150bp upstream cloned of *M. tuberculosis* *rv1378c* gene containing its SOS box region upstream of *lacZ* reporter plasmid and is resistant to kanamycin. | This study |
| *M. tuberculosis* H37Ra | Avirulent, high-efficiency transformation strain of  *M. tuberculosis.* | Laboratory stock |
| *M. tuberculosis* H37Rv | Virulent, high-efficiency transformation strain of  *M. tuberculosis.* | Laboratory stock |
| *S. aureus* ATCC 25923 | Isolate used as a standard laboratory testing control strain. | Laboratory stock |
| **Plasmids/constructs** | | |
| pET28a (+) | Expression vector (pBR322 ori), strong phage promoter (T7), IPTG induction (lac operon), and kanamycin selection (kan^r^). | Novagen |
| pSD5B | Mycobacterial replicative plasmid with *lacZ* reporter. The plasmid confers resistance to kanamycin. | Kind gift from  Dr. Vinay Nandicoori (CCMB) |
| *P_dnaE2_*~*mCherry* | 150bp upstream of *M. tuberculosis* *dnaE2* gene containing its SOS box region cloned upstream of *mCherry* between BamHI and NotI sites in pMV262~*mCherry* to create a SOS reporter plasmid resistant to kanamycin. | This study |
| pMV262~*mCherry* | Mycobacterial replicative plasmid with pAL5000 mycobacterial origin of replication. *mCherry* is constitutively expressed from *hsp* promoter which is cloned between BamHI and EcoRI sites. The plasmid confers resistance to kanamycin. | Kind gift from  Dr. Krishna Kurthkoti (RGCB, Trivandrum) |
| *P_rv1378c_*~*lacZ* | 150bp upstream of *M. tuberculosis* *rv1378c* gene containing its SOS box region cloned upstream of *lacZ* between SphI and XbaI sites in pSD5B~*lacZ to* create a SOS reporter plasmid resistant to kanamycin. | This study |
| S160A Mtb LexA | Catalytic site mutant of Mtb LexA which was used as a backbone for generating S160A/K197A  Mtb LexA. | Kind gift from  late Dr. M. Vijayan (1) (IISc, Bengaluru) |

**Table S3: Oligonucleotide primers used in this study**

| **Primer for** | **Forward Primer (FP) / Reverse Primer (RP) Sequences** |
| --- | --- |
| K197A Mtb LexA and  S160A/K197A Mtb LexA | FP- 5´ GGCCACCGTCgcGACGTTCAAACG 3´  RP- 5´ TCACCGTCGATCATGGCC 3´ |
| *dnaE2*_44mer | FP- 5´ Btn ACAACTGCGCTGTATCGAACAATTGTTCGATATACTGTGGAATG 3´  RP- 3´TGTTGACGCGACATAGCTTGTTAACAAGCTATATGACACCTTAC 5´ |

Btn- Biotinylated

**References:**

1. Chandran,A. V., Srikalaivani,R., Paul,A. and Vijayan,M. (2019) Biochemical characterization of Mycobacterium tuberculosis LexA and structural studies of its C-terminal segment. *Acta Crystallogr D Struct Biol*, **75**, 41–55.

2. Madhavi Sastry,G., Adzhigirey,M., Day,T., Annabhimoju,R. and Sherman,W. (2013) Protein and ligand preparation: Parameters, protocols, and influence on virtual screening enrichments. *J Comput Aided Mol Des*, **27**, 221–234.

3. Little,J.W. (1991) Mechanism of specific LexA cleavage: autodigestion and the role of RecA coprotease.

4. Friesner,R.A., Murphy,R.B., Repasky,M.P., Frye,L.L., Greenwood,J.R., Halgren,T.A., Sanschagrin,P.C. and Mainz,D.T. (2006) Extra precision glide: Docking and scoring incorporating a model of hydrophobic enclosure for protein-ligand complexes. *J Med Chem*, **49**, 6177–6196.

5. Halgren,T.A., Murphy,R.B., Friesner,R.A., Beard,H.S., Frye,L.L., Pollard,W.T. and Banks,J.L. (2004) Glide: a new approach for rapid, accurate docking and scoring. 2. Enrichment factors in database screening. *J Med Chem*, **47**, 1750–1759.

6. Friesner,R.A., Banks,J.L., Murphy,R.B., Halgren,T.A., Klicic,J.J., Mainz,D.T., Repasky,M.P., Knoll,E.H., Shelley,M., Perry,J.K., *et al.* (2004) Glide: a new approach for rapid, accurate docking and scoring. 1. Method and assessment of docking accuracy. *J Med Chem*, **47**, 1739–1749.

7. Greenwood,J.R., Calkins,D., Sullivan,A.P. and Shelley,J.C. (2010) Towards the comprehensive, rapid, and accurate prediction of the favorable tautomeric states of drug-like molecules in aqueous solution. *J Comput Aided Mol Des*, **24**, 591–604.

8. Zhu,K., Borrelli,K.W., Greenwood,J.R., Day,T., Abel,R., Farid,R.S. and Harder,E. (2014) Docking covalent inhibitors: A parameter free approach to pose prediction and scoring. *J Chem Inf Model*, **54**, 1932–1940.

9. Bowers,K.J., Chow,E., Xu,H., Dror,R.O., Eastwood,M.P., Gregersen,B.A., Klepeis,J.L., Kolossvary,I., Moraes,M.A., Sacerdoti,F.D., *et al.* (2006) Scalable algorithms for molecular dynamics simulations on commodity clusters. *Proceedings of the 2006 ACM/IEEE Conference on Supercomputing, SC’06*, 10.1145/1188455.1188544.

10. Roos,K., Wu,C., Damm,W., Reboul,M., Stevenson,J.M., Lu,C., Dahlgren,M.K., Mondal,S., Chen,W., Wang,L., *et al.* (2019) OPLS3e: Extending Force Field Coverage for Drug-Like Small Molecules. *J Chem Theory Comput*, **15**, 1863–1874.

11. Miller,J. (1972) Assay of β-galactosidase. Experiments in Molecular Genetcis. *Experiments in molecular genetics*.

12. Agrawal,P., Miryala,S. and Varshney,U. (2015) Use of Mycobacterium smegmatis Deficient in ADP-Ribosyltransferase as Surrogate for Mycobacterium tuberculosis in Drug Testing and Mutation Analysis. *PLoS One*, **10**, e0122076.
